# Editorial: Novel and potential markers for prediction of outcome in patients with acute and chronic coronary heart disease

**DOI:** 10.3389/fcvm.2019.00066

**Published:** 2019-05-22

**Authors:** Hugo ten Cate, Frederic Kontny, Dennis W. Nilsen

**Affiliations:** ^1^Laboratory for Clinical Thrombosis and Hemostasis, Department of Internal Medicine, Cardiovascular Research Institute Maastricht, Maastricht University Medical Center, Maastricht, Netherlands; ^2^Department of Cardiology, Stavanger University Hospital, Stavanger, Norway; ^3^Drammen Heart Center, Drammen, Norway; ^4^Department of Clinical Science, Faculty of Medicine, University of Bergen, Bergen, Norway

**Keywords:** prognosis, chronic heart diseases (CHD), biomarker, acute heart disease, atherosclerosis, biomarkers, acute coronary heartdisease

Cardiovascular disease (CVD) associated with atherosclerosis and/or atrial fibrillation form the main cause of death globally. Timely identification of patients at risk of CVD is therefore paramount. Through early screening of risk conditions, like hypertension, hypercholesterolemia, or diabetes, primary prevention aimed at correcting such risk factors can be implemented (1). Smoking cessation can also be pursued through intervention programs early on. However, optimal primary prevention is still limited; for instance, administration of aspirin to reduce the risk of thrombosis in *all* subjects of certain age is useless and potentially harmful (bleeding), as again reiterated by recent large-scale clinical prevention studies [commented on in Song and French (2)]. Recommending aspirin prophylaxis needs to be stratified according to additional risk factors, such as diabetes (3) or coronary artery calcification (4), to reduce the number needed to treat to prevent CVD manifestations to acceptable levels. Similar arguments can be provided for the use of statins, and previous attempts to provide additional pharmacological prophylaxis against CVD by the “polypill” concept failed to become implemented. Assessment of vascular dysfunction may help in selecting those at highest risk; we mentioned calcification, a strong marker of increased cardiovascular (CV) risk. In this thematic series, Bonarjee discusses the relevance of another marker of vascular dysfunction, arterial stiffness. This functional vascular marker is a predictor of CV events and mortality, independent of traditional risk factors for CV disease. Gradually, vascular functional assessment and imaging tools for calcification and unstable plaque characteristics appear to become part of the workup by cardiologists and other specialists of patients suspected of CVD.

There is still a substantial need for additional biomarkers that indicate CVD progression at a stage when, ideally, primary prevention would still be feasible. This topic has been addressed during later decades and reliable applicable biomarkers are still lacking in clinical practice. Blood biomarkers other than lipids do not go beyond additional C-reactive protein (CRP) measurement, but even this indicator of inflammation is not linked to specific therapeutic consequences. A recent clinical trial aimed at reducing inflammation in at-risk for CVD subjects only showed a modest protective effect upon lowering Il-6 levels (in those stratified for elevated CRP at baseline (5), but studies with methotrexate or colchicine, less specific anti-inflammatory drugs, were negative (6). Hence, determining CRP in all subjects of certain age or CVD risk profile does not seem warranted yet.

Does this mean that the laboratory biomarker perspective is bleak for CVD prediction? This does not seem to be the case. In fact, we may not yet have explored the possibilities of novel and promising biomarkers that include troponins for ischemic damage, natriuretic peptides, growth differentiation factor 15 (GDF-15) for cardiovascular stress and dysfunction, galectin-3 (fibrosis), cystatin (renal dysfunction) or specific cytokines (inflammation) or indices of coagulation activity (d-dimer) (7). In patients with acute coronary syndrome (ACS), NT-proBNP, and GDF-15 were found to be strong markers associated with all-cause death in a recent study (8).

In this theme series additional biomarker approaches have been reported, including circulating inflammation cell populations (Meeuwsen et al.; Gawdat et al.). Meeuwsen et al. review the importance of different leukocyte populations in relation to atherosclerosis and its complications. Whereas traditionally, leukocytosis was associated with inflammation and also with specific features of atherosclerosis, more recent research provided substantial evidence for the important roles of neutrophils, monocytes, and lymphocytes in distinct steps of the atherosclerosis process. Gwadat et al. provide in an original study, data on the neutrophil-to-lymphocyte ratio (NLR) and specific monocyte subsets (CD16) behavior following cardiac surgery. These shifts in cell subsets and function, part of the postoperative inflammatory response, may have consequences for the risks of CV events in such patients. For that reason, studies like the one from Gwadat et al. contribute knowledge that may ultimately have clinical consequences. What are such possible consequences? For one, modifying the course of inflammation by boosting the immune system could be a solution.

Naesgaard et al. studied the potential benefit of a high dose of purified omega-3 as compared to corn oil on vitamin D levels in patients after having survived an acute myocardial infarction. Previous work from this team led by Prof. Dennis Nilsen, showed that serum vitamin D levels were associated with total and CV mortality in patients with suspected acute chest syndrome (9). The present study did not establish any favorable effects of longterm omega-3 administered as a combination of eicosapentaenoic acid (EPA) and docosahexaenoic acid (DHA), on vitamin D levels. Given recent lack of evidence of any favorable effects of omega-3 supplementation at lower dose ranges on outcomes in subjects at risk of CVD, the influence on prognosis of this type of intervention may be limited, with no effect in healthy individuals with a high dietary background of omega-3, as recently reported in the VITAL study (10), whereas purified EPA given alon e in a dose of 4 g per day (twice the amount of EPA as compared to that of the present study by Naesgaard et al.) may exert a beneficial impact on prognosis, as demonstrated in the REDUCE-IT study (11). Furthermore, in the VITAL study, cardiovascular endpoints were unaffected by vitamin D supplementation (12), suggesting that lower values of vitamin D may be a result of established disease, rather than a causal factor.

Finally, two studies addressed different aspects of the coagulation system in patients with peripheral artery disease (PAD), a severe manifestation of atherosclerosis. In the hunt for biomarkers, Kleinegris et al. provide evidence in albeit a small case control study, for differences in clot properties, generated *ex vivo*. An apparent increase in whole blood clotting derived from PAD patients, possibly related to increased fibrinogen levels, provide an indication for hypercoagulability in such patients. These data support the notion that in peripheral artery disease (PAD) not just platelets are involved in driving atherothrombosis, but also clotting may contribute (13). Clot properties also were a critical determinant in patients with ACS (14). The latter lends support to some of the observed advantages of the so-called COMPASS regimen comprising low dose rivaroxaban (2.5 mg bd) combined with aspirin in lowering CV mortality in high risk CAD and PAD patients (15). The final study exploring a possible common thrombo-inflammatory network in patients with PAD vs. those who have suffered deep vein thrombosis (DVT), evaluates additional biomarkers for neutrophil activation and clotting (Kremers et al.). Although common drivers of thrombosis risk were not clearly detected in this study, it stimulates further research toward biomarkers that could pick up different stages of thrombo-inflammation in acute or chronic CVD.

Where does this leave us with respect to current biomarker status in CVD? According to experts like Lars Wallentin, there is much hope for this field and we are just starting to explore the potential (lecture at “Eurothrombosis”, Barcelona, 2018). Given the recent publications on above mentioned biomarkers, this may indeed be true. Combining several very promising markers of different elements of CVD (ischemia, necrosis, thrombo-inflammation, clot lysis, and fibrosis) may increase the diagnostic yield in patients at risk of CV complications and death. The fields of proteomics and genomics (e.g., miRNAs) are also moving and it is a matter of time before phenotyping patients based on biomarker cluster analysis will become clinically useful. How much time? That is anybody's guess, but we would say one decade ahead, biomarkers will be embedded in the diagnostic toolbox of most practicing physicians dealing with patients at risk of CVD.

## Author Contributions

All authors listed have made a substantial, direct and intellectual contribution to the work, and approved it for publication.

### Conflict of Interest Statement

The authors declare that the research was conducted in the absence of any commercial or financial relationships that could be construed as a potential conflict of interest.
